# Language Brain Representation in Bilinguals With Different Age of Appropriation and Proficiency of the Second Language: A Meta-Analysis of Functional Imaging Studies

**DOI:** 10.3389/fnhum.2019.00154

**Published:** 2019-05-21

**Authors:** Elisa Cargnelutti, Barbara Tomasino, Franco Fabbro

**Affiliations:** ^1^Scientific Institute, IRCCS E. Medea, Dipartimento/Unità Operativa Pasian di Prato, Udine, Italy; ^2^Cognitive Neuroscience Laboratory, DILL, University of Udine, Udine, Italy; ^3^PERCRO Perceptual Robotics Laboratory, Scuola Superiore Sant’Anna, Pisa, Italy

**Keywords:** meta-analysis, bilingualism, age of appropriation (AoA), proficiency, first language (L1), second language (L2)

## Abstract

Language representation in the bilingual brain is the result of many factors, of which age of appropriation (AoA) and proficiency of the second language (L2) are probably the most studied. Many studies indeed compare early and late bilinguals, although it is not yet clear what the role of the so-called critical period in L2 appropriation is. In this study, we carried out coordinate-based meta-analyses to address this issue and to inspect the role of proficiency in addition to that of AoA. After the preliminary inspection of the early (also very early) and late bilinguals’ language networks, we explored the specific activations associated with each language and compared them within and between the groups. Results confirmed that the L2 language brain representation was wider than that associated with L1. This was observed regardless of AoA, although differences were more relevant in the late bilinguals’ group. In particular, L2 entailed a greater enrollment of the brain areas devoted to the executive functions, and this was also observed in proficient bilinguals. The early bilinguals displayed many activation clusters as well, which also included the areas involved in cognitive control. Interestingly, these regions activated even in L1 of both early and late bilingual groups, although less consistently. Overall, these findings suggest that bilinguals in general are constantly subjected to cognitive effort to monitor and regulate the language use, although early AoA and high proficiency are likely to reduce this.

## Introduction

How does the brain of a bilingual person work? Research on this topic has extensively developed in the last decades, with an increasing number of studies devoted to the identification of the brain areas activated when bilinguals perform language tasks in the known languages. Bilinguals are different from each other in several respects. Given the definition of a bilingual as a person that masters more than one language or dialect (see Fabbro, 1999, 2001), it appears clear that this may apply to a wide range of individuals. For this reason, the bilinguals assessed in the published studies rarely form consistent and homogeneous groups. It follows that a comprehensive and universally accepted picture about the bilingual brain functioning is still lacking and that some questions are yet to be answered.

In this study, we tried to address some of these issues. The first notes the possibly different language brain representation in relation to the so defined *critical period*. This represents a specific age after which the learning process becomes challenging and the achieved performance in the second language (L2) hardly equals that of the native or first language (L1). In the past decades, an extensive debate has concerned the identification of the L2 *age of acquisition* or *age of appropriation* (as lately better defined, see Paradis, 2009) cutoff (hereafter, AoA). Some authors set it around puberty, a period during which language skills fully develop (e.g., Lenneberg, 1967; Long, 1990; Locke and Bogin, 2006), whereas others suggest the period around 6–7 years of age to be crucial, because, after this age, learning some linguistic skills becomes challenging (e.g., Johnson and Newport, 1989).

As long as AoA is judged as one of the parameters that mainly determine the L2 performance and shapes its brain representation, many studies compared the language networks between *early* and *late* bilinguals, hence between bilinguals having approached L2 either before or after the defined AoA cutoff. Although not univocally, most of these studies used 6 years of age as the AoA cutoff. This choice is motivated by the important developmental events taking place around this age. First, the brain is almost at its adult size (e.g., Giedd et al., 1999; Casey et al., 2000) and most of the myelination processes are complete (e.g., Nakagawa et al., 1998). Concerning language, skill achievement is attained in almost every domain, despite the fact that not all of the skills are perfectly mastered yet (e.g., Skeide and Friederici, 2016). Another important change concerns the memory systems supporting the cognitive processes. At this age, memory is organized as in adults and the verbal component takes on importance with respect to the visuo-spatial components (e.g., Gathercole et al., 2004).

Important changes also take place concerning the dissociation between implicit and explicit memory systems (see Paradis, 1994, 2004, 2009; Ullman, 2001, 2005, 2006). Up to this age, in fact, children acquire skills through implicit memory, therefore in an almost unconscious way. These skills are easily internalized and automatically applied. Along with development, this memory system becomes less flexible and late-learned skills are therefore mainly supported by explicit memory, with the enrollment of conscious brain processes. These skills are unlikely to become highly automatized, in particular concerning some language domains, such as grammar and phonology/articulation, whereas lexico-semantics appeared to be less affected by AoA (see also Ruben, 1997). In this case, the critical AoA seems to fall on adolescence, as long as the lexical knowledge mainly depends on the declarative memory capacity, then on proficiency and extent of use.

Another tricky aspect concerns the effects of the acquisition of both languages roughly simultaneously since birth. Such bilinguals are referred to as *simultaneous*, in comparison with the *sequential* bilinguals, who, irrespective of their AoA, had approached L2 successively to L1 and possibly when L1 acquisition was almost complete. As the majority of bilinguals belongs to the second category, neuroimaging data on the language brain networks in simultaneous bilinguals is reported in very few papers. Rather, studies more often include bilinguals having learned the two languages at least in the very first years of life (see Supplementary Table S1). In this sense, it would be interesting to inspect whether the language brain networks of *very early* bilinguals differ from those of general early bilinguals, as inspected in a few studies (see Supplementary Table S1).

A few previous meta-analyses focused on the functional networks associated with each language in the groups of early and late bilinguals. In this respect, Liu and Cao (2016) found that L2 activated several regions (i.e., insula and frontal cortex areas) more than L1 and this especially occurred in the group of late bilinguals. Similarly, Indefrey (2006), who conducted an explorative investigation of the areas that activate in bilinguals with different AoA, observed that it was more likely for individuals with late AoA to have an overall greater activation (especially in the left inferior frontal gyrus). The author reported a similar trend for bilinguals with low proficiency/exposure.

The aspect of language proficiency has been addressed in another meta-analysis (Sebastian et al., 2011). In this case, the authors observed that the L1 and L2 networks were more similar to each other in the group of the high-proficiency bilinguals, whereas greater differences between the two languages emerged as the result of low proficiency. Actually, evidence from the clinical literature seems to suggest that factors such as language proficiency and use/exposure are sometimes more relevant than mere AoA. There were indeed cases of bilingual aphasia in which the language that was premorbidly “weaker” was the most affected, whereas the language that the patient mastered better was less impaired. This indicated that proficiency in a given language is sometimes more relevant in predicting the impairment profile in bilingual aphasia (e.g., Edmonds and Kiran, 2006; Druks and Weekes, 2013; Gray and Kiran, 2013).

Nevertheless, a recently published systematic review on bilingual aphasia reported on the role of proficiency and use to be secondary to that of AoA (Kuzmina et al., 2019). Actually, L2 was more preserved—probably because of its stronger brain representation—in the case it was the best-mastered or mostly used language premorbidly, but only in early bilinguals; the effect of proficiency and use were instead limited for late bilinguals. In summary, although both AoA and proficiency appear to be relevant in shaping the bilingual brain, their relative role is not yet clear and the extent to which the proficiency level might scale down the role of AoA has not been yet investigated.

## The Current Meta Analysis

The principal aim of the present meta-analysis was to shed further light on the impact of AoA on the overall language brain representation and on those specifically associated with each language. Therefore, we tried to derive which brain regions bilinguals activate in a consistent way when performing language tasks in known languages. We carried out the analysis separately for the groups of early and late bilinguals. In addition, we wanted to inspect if some reliable activations could be found in a subgroup of very early bilinguals. After these more global analyses, we investigated the specific activations associated with each language, to then compare L1 and L2 within each group (i.e., early and late bilinguals), and L1s and L2s between groups. Lastly, we aimed to investigate the effect of proficiency. In particular, we inspected whether the two language networks in early and late bilinguals differed as the result of different proficiency levels.

With respect to the previous meta-analyses, this study: (i) explored more in depth the language networks associated with each language as the result of AoA first and of proficiency, second; (ii) investigated the language brain representation resulting from a very early L2 acquisition; and (iii) adopted quite stringent criteria for both paper inclusion and data analysis, in order to ascertain the strength of the resultant findings.

We hypothesized to confirm the results from previous meta-analyses in terms of an overall greater functional activation for the group of the late bilinguals with respect to that of the early bilinguals. Regarding the comparison between the two languages, we attended greater functional activation for L2 than L1 and expected to find this difference even in the group of early bilinguals. Finally, we expected the differences between early and late bilinguals and between L2 and L1 to reduce in high vs. low proficient bilinguals.

## Materials and Methods

### Paper Search and Selection

**Figure 1:**
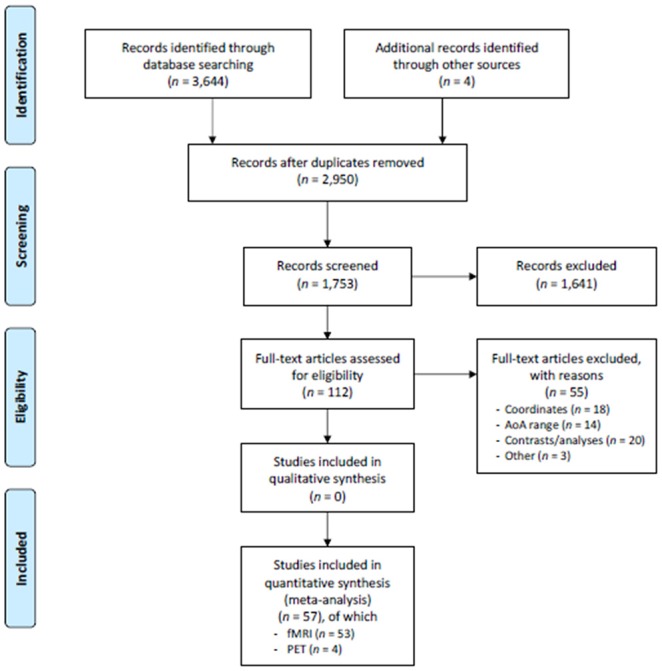
PRISMA flowchart. Schematic representation of the paper search and selection process. From Moher et al. (2009).

In the current meta-analysis, we included the papers selected from the pool of English-written articles published between 1995 and the end of 2016. To be included, the papers had to report neuroimaging studies (by fMRI or PET) involving healthy adult participants (aged 18–60). We performed the research in MedLine and Scholar databases, using keywords such as “fMRI” or “functional MRI,” “PET,” “bilingual*.” The sample was further integrated with some papers found by inspecting the list of references of the papers resulting from this research. The paper selection procedure is sketched out in the PRISMA flow chart (Moher et al., 2009) in Figure 1.

In the selection, we excluded cases of bimodal bilingualism (i.e., with one of the languages being a sign language) and studies assessing the language abilities specific to bilingualism, such as translation/interpretation and switching. We hence restricted the selection to the studies having addressed the main structural domains (i.e., lexico-semantics, phonology/articulation, and morpho-syntax) and we excluded those investigating more specific tasks, as the affective/emotional components of language (e.g., role of emotional words) or numbers and mathematics. The selection was not limited to specific language families. We were confident in including different language tasks from different languages in the same analysis, given that the algorithm we used (see afterwards) looks for the areas showing a convergence of activation across different experiments and therefore provides only consistently recurring activations. Finally, in order to reduce confounding effects, we also excluded studies performing assessments after learning/training processes (e.g., learning of new words or grammar rules, training in a barely mastered language) or after some manipulations to language exposure. Further, the participant samples in these studies were normally gender-balanced and quite homogeneous in terms of age (often including young adults). This assured more robust findings.

For this analysis, we included only the studies that have been published after a peer-reviewed process. In this sense, the study might be subjected to a publication bias. Nevertheless, coordinate-based meta-analyses differ from effect-size meta-analyses in that they look for the spatial convergence between the reported coordinates; hence, they do not quantify the effect size, which is prone to bias. Therefore, this analysis seems to be less susceptible to region- and task-dependent biases and was not affected by the lacking inclusion of unpublished data (see Fox et al., 1998; Rottschy et al., 2012). Moreover, to reduce other sources of bias, we included only the results from whole-brain analysis, excluding those resulting from *a priori* selected ROIs.

This first selection resulted in 112 papers, which we further scrutinized to obtain the final sample. This selection was followed by the exclusion of some additional papers due to: (i) absent or incomplete (not full 3D) coordinates, including only coordinate ranges, coordinates that were reported only for single subjects, coordinates from *a priori* selected ROIs (not derived from the observed activations), *n* = 19; (ii) analyses where the contrasts were not informative (e.g., they did not differentiate between different languages or between bilinguals and monolinguals), were too specific or regarded a very low level of linguistic processing (e.g., passive viewing of single letters), *n* = 17; (iii) AoA that was not explicitly reported or did not fit our classification (see afterwards) *n* = 20; and (iv) other reasons (e.g., tasks assessing a linguistic ability “contaminated” by another aim, such as reading finalized to memorization), *n* = 3.

To define the two groups of early and late bilinguals, we adopted the age of 6 years as the AoA cutoff. The previously mentioned developmental steps occurring around this age guided our choice, further supported by the high number of studies having adopted this same cutoff. In fact, most of the studies classified bilinguals in early and late following an AoA that was, respectively, below and above 6 years of age. Alternatively, studies focused on either group, therefore either on early bilinguals, for instance from bilingual communities, or on late bilinguals, typically represented by people having learnt L2 at school. Concerning the very early bilinguals, an inspection of the studies we selected led us to include those with participants having an AoA up to 3 years. Unfortunately, the paucity of the studies on the early bilinguals that acquired L2 after L1 did not allow a specific analysis on this subgroup.

To meet the specific purposes of our paper, we, therefore, excluded the studies where AoA was not explicitly indicated or the reported AoA did not allow to include the participants in the groups we defined on the basis of the selected AoA cutoff (*n* = 17). Finally, in order to reduce additional sources of variability, we also excluded studies that investigated language learning in adulthood (*n* = 3).

Concerning proficiency, we observed that many studies reported self-rating assessments, or a general evaluation based on the performance in a single task (e.g., naming). Only a small percentage of studies reported a quantitative assessment by structured tests (e.g., TOEFL test for the English language). These ratings did not allow to reliably classify bilinguals from the proficiency viewpoint. Nevertheless, the studies in which the participants achieved a high score in a comprehensive language assessment or were defined to have a high proficiency, were, more consistently represented than those with low or intermediate proficiency. For this reason, we limited the analysis to the subsample of high proficient bilinguals and excluded from this subgroup the bilinguals whose proficiency in L2 was greater than in L1, in order to remove potential confounds.

The process of paper selection was preceded by the definition, by the three authors, of the objective criteria for study inclusion and exclusion. During the process, we consulted with one other to define additional criteria based on the issues that emerged in the meanwhile. At the end of the process, we discussed together about the residual papers that we did not know whether to include or not. In this way, we assured a consistent, unbiased selection procedure.

The final sample consisted of 57 papers (53 fMRI and four PET studies), from which we identified the groups of early bilinguals (74 experiments; 536 foci; 1,048 subjects), very early bilinguals (17 experiments; 91 foci; 227 subjects), and late bilinguals (174 experiments; 1,351 foci; 2,519 subjects), see Supplementary Table S1 for paper list details.

### Statistical Analyses

We carried out the meta-analyses using the coordinate-based activation likelihood estimation (ALE) algorithm developed for neuroimaging data (e.g., Turkeltaub et al., 2002; Eickhoff et al., 2009; Laird et al., 2009a,b). The algorithm looks for convergence across the experiment data, by evaluating whether the clustering is higher than that expected under the null distribution of a random spatial association. It, therefore, treats the reported foci as centers for 3D Gaussian probability distributions, to capture the spatial uncertainty associated with each focus. The provided probability distribution maps, which were weighted on the number of subjects in each study, described the probability for a given focus to lay within a given voxel.

We thresholded the probability maps for the main effect analyses at *p* < 0.05 (cluster-level corrected for multiple comparisons) and set a minimum cluster size to 200 mm^3^ (25 voxels). For the analysis on individual languages, we reduced the extent threshold to 120 mm^3^ (15 voxels). For the contrast analyses, we used threshold values of *p* < 0.001 (uncorrected) and a minimum cluster size of 80 mm^3^ (10 voxels). Nevertheless, for the conjunction results, we retained only a minimum of 120-mm^3^ (15 voxels) clusters, in order to exclude a possible incidental overlap between the ALE maps from individual analyses (see Rottschy et al., 2012).

We performed the following analyses:

(i) overall language brain representation in early, very early, and late bilinguals.
  In the first preliminary analysis, we investigated the overall (not language-specific) functional brain representation of early and late bilinguals (main effects). We also performed the same analysis on a subgroup of early bilinguals that have acquired the two languages roughly simultaneously (up to the age of 3) and therefore defined as very early bilinguals.
(ii) L1 and L2 networks and between-language and between-group comparisons.
  We then focused on the functional networks associated with each language. We performed the analysis separately for late and early bilinguals, excluding the very early bilinguals for whom a distinction between L1 and L2 based on the AoA was not possible. We first carried out the main effect analyses; next, we performed between-group analyses to compare the networks of L1s and L2s across the two groups and within-group analysis to compare the functional networks associated with L1 and L2 within each group.
(iii) L1 and L2 networks in proficient bilinguals and between-language and between-group comparisons.
  We replicated the recently mentioned analyses on a subgroup of proficient bilinguals.

We reported the coordinates in the Montreal Neurological Institute (MNI) standard space. The coordinates that were standardized to the Talairach and Tournoux (1988) space in the included studies were converted to the MNI space by the icbm_spm2tal transform. To define the precise anatomical localization and label of the resulting areas, we used the SPM Anatomy toolbox (Eickhoff et al., 2005), running on MATLAB. We, therefore, reported the macro-anatomic localization and, when provided, the cytoarchitectonic location.

## Results

### Whole Language Brain Representation in Early, Very Early, and Late Bilinguals

**Figure 2:**
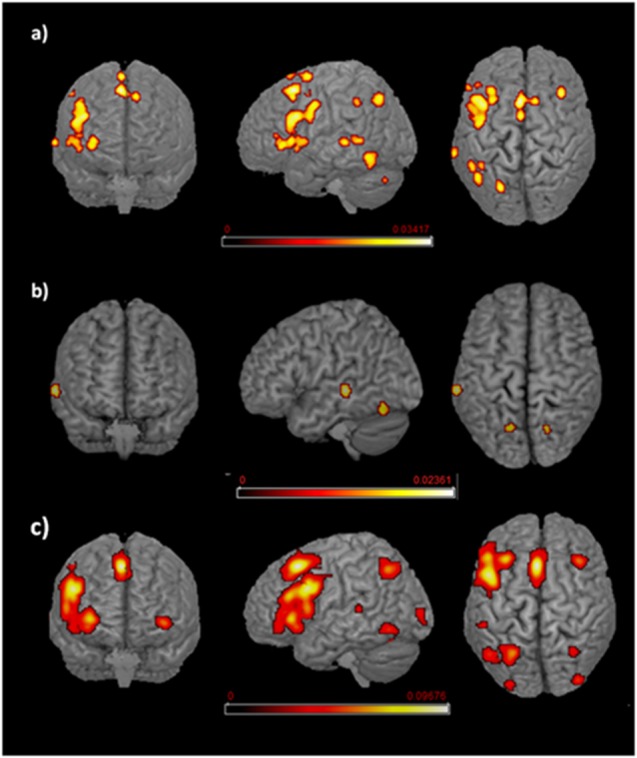
Language networks associated with different age of appropriation (AoA). Rendered templates of the main effect analysis results for **(A)** early bilinguals, **(B)** very early bilinguals, and **(C)** late bilinguals. Color bars indicate the activation likelihood estimation (ALE) values.

The main effect results for each group are reported in Tables 1.1, 1.2 and Figure 2.

**Table 1.1:** Main effect results of the activation likelihood estimation (ALE) meta-analysis for the groups of early and very early bilinguals.

Cluster (area)		MNI coordinates			Cluster size (voxels)	Extrema value
		*x*	*y*	*z*		
**Early bilinguals**						
1	L inferior parietal lobule	−24	−68	44	71	0.029
2	L inferior occipital (FG4)	−44	−58	−12	148	0.030
3	L middle temporal gyrus	−50	−48	4	41	0.025
5	L middle temporal gyrus (TE3)	−68	−32	2	152	0.037
6	R middle temporal gyrus (TE3)	70	−14	−8	53	0.029
7	L precentral gyrus	−46	−6	38	631	0.033
	L inferior frontal gyrus	−40	12	28		0.033
	L inferior frontal gyrus	−46	18	22		0.031
	L precentral gyrus	−42	0	28		0.027
8	L precentral gyrus	−52	2	50	25	0.026
9	L posterior-medial frontal gyrus	−2	2	66	264	0.036
	L posterior-medial frontal gyrus	−4	16	52		0.034
10	L rolandic operculum	−48	8	2	114	0.024
	L insula	−44	12	−4		0.022
	L inferior frontal gyrus (BA 44)	−56	8	10		0.021
11	R posterior-medial frontal gyrus	12	16	46	36	0.027
12	L insula	−30	18	4	124	0.028
	L insula	−32	26	0		0.027
13	R insula	36	24	−4	59	0.031
14	L inferior frontal gyrus (BA 45)	−54	30	4	137	0.032
15	R cerebellum (lobule VIIa, crus I)	36	−74	−28	25	0.023
**Very early bilinguals**						
1	L middle temporal gyrus	−66	−30	−2	46	0.020
2	L cerebellum (lobule VI)	−16	−68	−20	53	0.020
3	R cerebellum (lobule VIIa, crus I)	18	−68	−16	45	0.018

*Note. For the anatomical localization the macro-anatomic area is indicated and, when provided, the cytoarchitectonic location is indicated (in parentheses). Analyses were performed with a threshold of *p* < 0.05 (corrected) and extent threshold of 25 voxels. FG4, Fusiform area; hIP2, Horizontal tracts of the intraparietal sulcus; IPS, Intraparietal sulcus; TE3, Higher auditory cortex*.

**Table 1.2:** Main effect results of the ALE meta-analysis for the group of the late bilinguals.

Cluster (area)		MNI coordinates			Cluster size (voxels)	Extrema value
		*x*	*y*	*z*		
**Late bilinguals**						
1	L middle occipital gyrus (hOc4lp)	−28	−92	4	83	0.039
2	R middle occipital gyrus (hOc4lp)	36	−88	8	70	0.050
3	L inferior occipital gyrus (FG4)	−46	−64	−12	180	0.049
4	L superior parietal lobule	−26	−64	46	422	0.063
5	R angular gyrus (IPS, hIP3)	30	−62	48	63	0.039
6	L middle temporal gyrus	−52	−36	8	30	0.037
7	L inferior frontal gyrus	−44	12	28	2,630	0.091
	L insula	−32	26	−2		0.071
	L inferior frontal gyrus (BA 45)	−48	28	18		0.070
	L inferior frontal gyrus (BA 44)	−54	12	10		0.062
	L inferior frontal gyrus	−50	32	10		0.047
	L inferior frontal gyrus (pars orbitaris)	−48	38	−8		0.039
	L precentral gyrus	−50	10	50		0.039
	L precentral gyrus	−52	2	50		0.033
	L precentral gyrus	−48	−4	40		0.030
8	L posterior-medial frontal gyrus	−2	20	48	842	0.096
9	R insula	36	24	−4	159	0.058
10	R cerebellum (lobule VIIa, crus I)	36	−74	−26	25	0.035
11	R cerebellum (lobule VI)	22	−66	−22	43	0.037

*Note. For the anatomical localization the macro-anatomic area is indicated and, when provided, the cytoarchitectonic location is indicated (in parentheses). Analyses were performed with a threshold of *p* < 0.05 (corrected) and extent threshold of 25 voxels. FG4, Fusiform area; hIP2, hIP3, Horizontal tracts of the intraparietal sulcus; hOc4lp, Lateral occipital cortex; IPS, Intraparietal sulcus*.

#### Early Bilinguals

With regard to the early bilinguals, functional activations emerged in the following regions of the left hemisphere: (i) inferior parietal lobule (including the intraparietal sulcus—area hIP2); (ii) inferior occipital gyrus (i.e., fusiform area); (iii) precentral gyrus; (vi) rolandic operculum; and (v) inferior frontal gyrus (i.e., BA 44, BA 45, and the dorsolateral prefrontal cortex, DLPFC); right-sided activations included (vi) the cerebellum (lobule VIIa and crus I) and bilateral activations; (vii) middle temporal gyri (including the higher auditory cortex—area TE3); (viii) posterior-medial frontal gyri; and (ix) the insulae.

#### Very Early Bilinguals

The very early bilinguals displayed activation, in the left hemisphere, of the (i) middle temporal gyrus and, in both the hemispheres, of the (ii) cerebella (lobule VI, in the left hemisphere; lobule VIIa and crus I, in the right hemisphere).

#### Late Bilinguals

The late bilinguals’ activation clusters included the following regions of the left hemisphere: (i) the inferior occipital gyrus (fusiform gyrus—area FG4); (ii) superior parietal lobule; (iii) middle temporal gyrus; (iv) precentral gyrus; (v) posterior-medial frontal gyrus; and (vi) inferior frontal gyrus (including BA 44, pars orbitaris, and DLPFC); activation clusters were also found in right (viii) angular gyrus (more precisely the intraparietal sulcus—area hIP3); and (ix) cerebellum (lobule VI, lobule VIIa, and crus I) and in bilateral (x) middle occipital gyrus (lateral cortex—area hOc4lp); and (xi) insulae.

### L1 and L2 Networks in Early and Late Bilinguals

**Figure 3:**
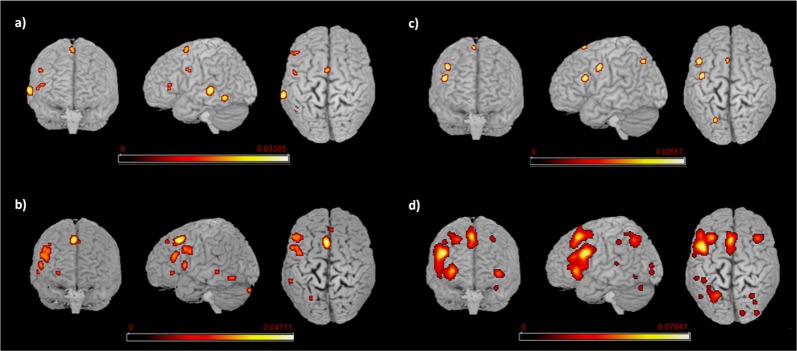
Language networks associated with L1 and L2 in the two groups of early and late bilinguals. Rendered templates of the main effect results for **(A)** early bilinguals’ L1; **(B)** late bilinguals’ L1; **(C)** early bilinguals’ L2; **(D)** late bilinguals’ L2. Color bars indicate the ALE values.

The functional brain activations associated with either L1 or L2 are detailed in Tables s 2, 3 for L1 and L2, respectively and are all represented in Figure 3.

**Table 2:** Results of the single ALE meta-analysis on L1 in the two groups of early and late bilinguals.

Cluster (area)		MNI coordinates			Cluster size (voxels)	Extrema value
		*x*	*y*	*z*		
**L1: Early bilinguals**						
1	L inferior temporal gyrus (FG4)	−46	−56	−12	58	0.027
2	L middle temporal gyrus (TE3)	−68	−34	2	107	0.034
3	L precentral gyrus	−50	0	32	15	0.020
4	L posterior-medial frontal gyrus	−2	4	64	35	0.024
5	L inferior frontal gyrus (BA 45)	−54	30	4	22	0.020
6	L inferior frontal gyrus	−46	32	10	58	0.020
**L1: Late bilinguals**						
1	L inferior occipital gyrus (FG4)	−46	−64	−12	400	0.028
2	L middle temporal gyrus	−58	−40	−2	184	0.027
	L precentral gyrus	−44	2	30		0.029
3	L precentral gyrus	−48	10	34	1,288	0.028
4	L inferior frontal gyrus (BA 44)	−54	10	8	424	0.029
5	L posterior-medial frontal gyrus	−4	20	48	1,504	0.047
6	L inferior frontal gyrus (BA 45)	−52	26	24	1,224	0.030
	L inferior frontal gyrus	−48	28	20		0.030
7	L insula	−28	28	−2	136	0.025
8	R superior medial gyrus	4	38	46	152	0.024
9	R cerebellum (lobule VIIa, crus I)	16	−90	−30	216	0.030

*Note. For the anatomical localization the macro-anatomic area is indicated and, when provided, the cytoarchitectonic location is indicated (in parentheses). Analyses were performed with a threshold of *p* < 0.05 (corrected) and extent threshold of 15 voxels. FG4, fusiform area; TE3, higher auditory cortex*.

**Table 3:** Results of the single ALE meta-analysis on L2 in the two groups of early and late bilinguals.

Cluster (area)		MNI coordinates			Cluster size (voxels)	Extrema value
		*x*	*y*	*z*		
**L2: Early bilinguals**						
1	L superior parietal lobule	−22	−70	46	22	0.023
2	L precentral gyrus	−42	−4	38	56	0.026
3	L inferior frontal gyrus	−48	18	22	56	0.027
4	L posterior-medial frontal gyrus	−4	20	66	18	0.022
**L2: Late bilinguals**						
1	R calcarine gyrus (V1)	14	−88	−2	32	0.027
2	R middle occipital gyrus (hOc4lp)	36	−88	8	29	0.028
3	R angular gyrus	28	−62	48	36	0.027
4	L superior parietal lobule	−24	−62	46	369	0.046
5	L inferior parietal lobule	−44	−40	42	31	0.029
6	L superior temporal gyrus	−54	−36	10	17	0.026
7	L inferior frontal gyrus	−46	12	26	2,212	0.078
	L insula	−32	26	−2		0.056
	L inferior frontal gyrus (BA 45)	−48	30	20		0.043
	L inferior frontal gyrus (BA 45)	−52	22	2		0.030
	L inferior frontal gyrus	−50	32	6		0.029
	L inferior frontal gyrus (pars orbitalis)	−52	24	−8		0.025
	L posterior-medial frontal gyrus	−2	22	50	815	0.062
8	L superior medial gyrus	−4	28	40		0.042
	L posterior-medial frontal gyrus	−4	8	54		0.037
9	R insula	38	24	−6	178	0.046
10	R cerebellum (lobule VIIa, crus I)	34	−72	−28	21	0.026

*Note. For the anatomical localization the macro-anatomic area is indicated and, when provided, the cytoarchitectonic location is indicated (in parentheses). Analyses were performed with a threshold of *p* < 0.05 (corrected) and extent threshold of 15 voxels. hOc4lp, lateral occipital cortex; V1, primary visual cortex*.

#### L1

Early bilinguals, when performing tasks in their L1, activated the following regions, all located in the left hemisphere: (i) the inferior temporal gyrus (fusiform gyrus); (ii) middle temporal gyrus (area TE3); (iii) precentral gyrus; (iv) posterior-medial frontal gyrus; and (v) the inferior frontal gyrus (Broca’s area—BA 45—and a region associable with the DLPFC).

The L1 activation clusters in late bilinguals included the following regions of the left hemisphere: (i) inferior occipital gyrus (fusiform gyrus—area FG4); (ii) middle temporal gyrus; (iii) precentral gyrus; (iv) posterior-medial frontal gyrus; (v) inferior frontal gyrus (including BA 44, BA 45, and DLPFC); and (vi) insula; right-sided activations were found in the (vii) superior-medial gyrus; and (viii) cerebellum (lobule VIIa and crus I).

#### L2

The activation clusters associated with early bilinguals’ L2 emerged in the following regions, all in the left hemisphere: (i) the superior parietal lobule; (ii) precentral gyrus; (iii) inferior frontal gyrus (region including the DLPFC); and (iv) the posterior-medial frontal gyrus.

The late bilinguals’ L2 functional activations were located in the following regions of the left hemisphere: (i) superior parietal lobule; (ii) inferior parietal lobule; (iii) superior temporal gyrus,; (iv) posterior-medial frontal gyrus; (v) inferior frontal gyrus (including BA 45, pars oribitaris, and DLPFC); and (vi) superior-medial gyrus; in the right hemisphere, activations emerged in (vii) calcarine gyrus (hOc1, V1); (viii) middle occipital gyrus (lateral cortex-area hOc4lp); (ix) angular gyrus; and (x) cerebellum (lobule VIIa and crus I); bilateral activations were observed in the (xi) insulae.

#### Between-Group Comparison Between L1s and L2s

**Figure 4:**
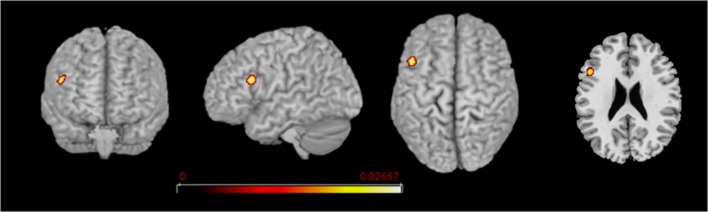
L2 network comparison between the groups of early and late bilinguals. Rendered templates and axial projection of the conjunction analysis results for L2: Early bilinguals ∩ Late bilinguals. Color bars indicate the *Z*-score values.

The activation clusters resulting from the between-group contrast conditions (i.e., comparison between L1s and L2s across the two groups of early and late bilinguals) are reported in Table 4 and Figure 4.

**Table 4:** Results of the between-group ALE meta-analysis for L1s and L2s.

Cluster (area)	MNI coordinates			Cluster size (voxels)	Extrema value
	*x*	*y*	*z*		
**L1s**					
Early bilinguals ∩ Late bilinguals					
No suprathreshold clusters of activation					
Early bilinguals > Late bilinguals					
No suprathreshold clusters of activation					
Late bilinguals > Early bilinguals					
No suprathreshold clusters of activation					
**L2s**					
Early bilinguals ∩ Late bilinguals					
1 L inferior frontal gyrus	−48	18	22	56	0.027
Early bilinguals > Late bilinguals					
No suprathreshold clusters of activation					
Late bilinguals > Early bilinguals					
No suprathreshold clusters of activation					

*Note. For the anatomical localization the macro-anatomic area is indicated and, when provided, the cytoarchitectonic location is indicated (in parentheses). Analyses were performed with a threshold of *p* < 0.001 (uncorrected) and 15 voxels for the conjunction analysis, and *p* < 0.001 (uncorrected) and 10 voxels for the subtraction analyses*.

##### L1: Conjunction (Early Bilinguals ∩ Late Bilinguals) and Subtraction Analyses (Early Bilinguals > Late Bilinguals and Late Bilinguals > Early Bilinguals)

No one area appeared to be consistently activated for L1 in conjunction of the two groups or in one group more than in the other.

##### L2: Conjunction (Early Bilinguals ∩ Late Bilinguals) and Subtraction Analyses (Early Bilinguals > Late Bilinguals and Late Bilinguals > Early Bilinguals)

Concerning L2, the areas activated in conjunction by the two groups were located in the left (i) inferior frontal gyrus (at the border between BA44 and DLPFC). The direct comparison did not show any clusters activating more consistently in either group over the other.

#### Within-Group Comparison Between L1 and L2

Results of the within-group comparison are reported in Table 5.

**Table 5:** Results of the within-group contrast ALE meta-analysis between L1 and L2 in the two groups of early and late bilinguals.

Cluster (area)	MNI coordinates			Cluster size (voxels)	Extrema value
	*x*	*y*	*z*		
**Early bilinguals**					
**L1 ∩ L2**					
No suprathreshold clusters of activation					
**Early bilinguals, L1 > L2**					
No suprathreshold clusters of activation					
**Early bilinguals, L2 > L1**					
No suprathreshold clusters of activation					
**Late bilinguals**					
**L1 ∩ L2**					
1 L precentral gyrus	−48	10	34	67	0.029
2 L precentral gyrus	−44	2	30		0.029
L posterior-medial frontal gyrus	−4	20	48	118	0.047
3 L inferior frontal gyrus (BA 45)	−52	26	24	74	0.030
L inferior frontal gyrus	−48	28	20		0.030
** L1 > L2**					
No suprathreshold clusters of activation					
** L2 > L1**					
1 L inferior frontal gyrus	−44	14	20	39	n/a
L inferior frontal gyrus	−43	12	24		n/a
2 L posterior-medial frontal gyrus	−2	20	58	17	n/a

*Note. For the anatomical localization the macro-anatomic area is indicated and, when provided, the cytoarchitectonic location is indicated (in parentheses). Analyses were performed with a threshold of *p* < 0.001 (uncorrected) and 15 voxels for the conjunction analysis, and *p* < 0.001 (uncorrected) and 10 voxels for the subtraction analyses*.

##### Early Bilinguals: Conjunction (L1 ∩ L2) and Subtraction (L1 > L2 and L2 > L1) Analyses

For the early bilinguals’ group, neither the conjunction nor the subtraction analysis provided suprathreshold activation clusters in the comparison between L1 and L2.

##### Late Bilinguals: Conjunction (L1 ∩ L2) and Subtraction (L1 > L2 and L2 > L1) Analyses

The late bilinguals activated the following left-hemisphere areas in conjunction with the two languages: (i) the precentral gyrus; (ii) posterior-medial frontal gyrus; and (iii) the inferior frontal gyrus (BA 45 and DLPFC).

The direct comparison between the two languages did not reveal any single region to be more consistently activated in L1 than in L2. Conversely, L2, when compared to L1, engaged more consistently in following regions, both in the left hemisphere: (i) the inferior frontal gyrus (region including the DLPFC); and (ii) the posterior-medial frontal gyrus.

### L1 and L2 Networks in Proficient Bilinguals

**Figure 5:**
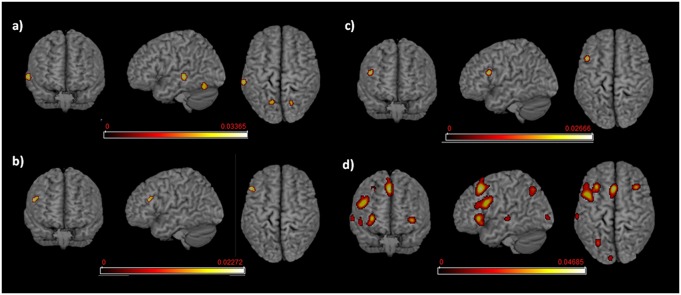
Language networks associated with L1 and L2 in the groups of proficient early and late bilinguals. Rendered templates of the main effect results for **(A)** proficient early bilinguals’ L1; **(B)** proficient late bilinguals’ L1; **(C)** proficient early bilinguals’ L2; **(D)** proficient late bilinguals’ L2. Color bars indicate the ALE values.

We re-ran the previous analyses on a subgroup of highly proficient bilinguals (16 studies including the early bilinguals, 17 studies including the late bilinguals). The functional networks associated with either L1 or L2 are detailed in Tables 6.1, 6.2 for L1 and L2, respectively and are represented in Figure 5.

**Table 6.1:** Results of the single ALE meta-analysis on L1 in the two groups of proficient early and late bilinguals.

Cluster (area)		MNI coordinates			Cluster size (voxels)	Extrema value
		*x*	*y*	*z*		
**L1: Proficient early bilinguals**						
1	L middle temporal gyrus (TE3)	−68	−34	2	113	0.036
2	L posterior-medial frontal gyrus	−2	4	66	19	0.023
**L1: Proficient late bilinguals**						
1	L inferior frontal gyrus (BA 45)	−52	28	24	48	0.023

*Note. For the anatomical localization the macro-anatomic area is indicated and, when provided, the cytoarchitectonic location is indicated (in parentheses). Analyses were performed with a threshold of p < 0.05 (corrected) and extent threshold of 15 voxels. TE3, Higher auditory cortex*.

**Table 6.2:** Results of the single ALE meta-analysis on L2 in the two groups of proficient early and late bilinguals.

Cluster (area)		MNI coordinates			Cluster size (voxels)	Extrema value
		*x*	*y*	*z*		
**L2: Proficient early bilinguals**						
1	L inferior frontal gyrus	−48	18	22	56	0.027
**L2: Proficient late bilinguals**						
1	L calcarine gyrus (hOc1)	−8	−96	0	26	0.023
2	L inferior parietal lobe	−28	−70	48	120	0.028
3	L middle temporal gyrus (TE3)	−66	−24	−2	31	0.023
4	L caudate nucleus	−8	8	0	16	0.022
5	L inferior frontal gyrus	−46	12	26	549	0.040
	L inferior frontal gyrus (BA 45)	−50	26	22		0.026
6	L posterior-medial frontal gyrus	−2	22	50	519	0.047
	L posterior-medial frontal gyrus	−4	20	66		0.023
7	L inferior frontal gyrus	−52	24	−8	37	0.023
8	L insula	−32	26	−2	230	0.038
9	R caudate nucleus	12	18	0	15	0.021
10	R insula	38	26	−4	102	0.035

*Note. For the anatomical localization the macro-anatomic area is indicated and, when provided, the cytoarchitectonic location is indicated (in parentheses). Analyses were performed with a threshold of *p* < 0.05 (corrected) and extent threshold of 15 voxels. hOc1, Primary visual cortex (V1); TE3, Higher auditory cortex*.

#### L1 in Proficient Bilinguals

For the early bilinguals, the functional activations associated with L1 emerged in the left: (i) middle temporal gyrus (area TE3—higher auditory cortex); and (ii) the posterior-medial frontal gyrus.

In the late bilinguals, the functional network included the left: (i) posterior-medial frontal gyrus; and (ii) the inferior frontal gyrus (BA 45).

#### L2 in Proficient Bilinguals

The early bilinguals’ L2 significantly activated a portion of the left (i) inferior frontal gyrus (region including the DLPFC).

The late bilinguals’ functional activations associated with L2 included different areas in the left hemisphere: (i) inferior parietal cortex; (ii) inferior frontal gyrus (including BA 45 and a region associable with the DLPFC); and (iii) posterior-medial frontal gyrus; bilateral activation was found in the (iv) caudate nuclei and (v) insulae.

#### Between-Group Comparison Between Early and Late Proficient Bilinguals

Results of the between-group comparison are reported in Table 6.3.

**Table 6.3:** Results of the between-group ALE meta-analysis for L1s and L2s in proficient bilinguals.

Cluster (area)	MNI coordinates			Cluster size (voxels)	Extrema value
	*x*	*y*	*z*		
**Proficient bilinguals’ L1s**					
Early bilinguals ∩ Late bilinguals					
No suprathreshold clusters of activation					
Early bilinguals > Late bilinguals					
No suprathreshold clusters of activation					
Late bilinguals > Early bilinguals					
No suprathreshold clusters of activation					
**Proficient bilinguals’ L2s**					
Early bilinguals ∩ Late bilinguals					
L inferior frontal gyrus	−48	18	22	56	0.027
Early bilinguals > Late bilinguals					
No suprathreshold clusters of activation					
Late bilinguals > Early bilinguals					
No suprathreshold clusters of activation					

*Note. For the anatomical localization the macro-anatomic area is indicated and, when provided, the cytoarchitectonic location is indicated (in parentheses). Analyses were performed with a threshold of *p* < 0.001 (uncorrected) and 15 voxels for the conjunction analysis, and *p* < 0.001 (uncorrected) and 10 voxels for the subtraction analyses*.

##### L1: Conjunction (Early Bilinguals ∩ Late Bilinguals) and Subtraction (Early Bilinguals > Late Bilinguals and Late Bilinguals > Early Bilinguals) Analyses

For L1, neither the conjunction nor the subtraction analysis provided suprathreshold activation clusters in the comparison between L1 and L2.

##### L2: Conjunction (Early Bilinguals ∩ Late Bilinguals) and Subtraction (Early Bilinguals > Late Bilinguals and Late Bilinguals > Early Bilinguals) Analyses

For L2, the conjunction analysis provided a shared activation cluster between early and late bilinguals in the left (i) inferior frontal gyrus (region including the DLPFC).

In the subtraction analyses, suprathreshold activation clusters did not result from either comparison.

#### Within-Group Comparison Between L1 and L2 in Proficient Bilinguals

Results of the within-group comparison are reported in Table 6.4.

**Table 6.4:** Results of the within-group ALE meta-analysis for L1s and L2s in proficient bilinguals.

Cluster (area)	MNI coordinates			Cluster size (voxels)	Extrema value
	*x*	*y*	*z*		
**Proficient early bilinguals**					
L1 ∩ L2					
No suprathreshold clusters of activation					
Early bilinguals, L1 > L2					
No suprathreshold clusters of activation					
Early bilinguals, L2 > L1					
No suprathreshold clusters of activation					
**Proficient late bilinguals**					
L1 ∩ L2					
1 L inferior frontal gyrus (BA 45)	−54	28	20	32	0.023
L1 > L2					
No suprathreshold clusters of activation					
L2 > L1					
No suprathreshold clusters of activation					

*Note. For the anatomical localization the macro-anatomic area is indicated and, when provided, the cytoarchitectonic location is indicated (in parentheses). Analyses were performed with a threshold of *p* < 0.001 (uncorrected) and 15 voxels for the conjunction analysis, and *p* < 0.001 (uncorrected) and 10 voxels for the subtraction analyses*.

##### Early Proficient Bilinguals: Conjunction (L1 ∩ L2) and Subtraction (L1 > L2 and L2 > L1) Analyses

For the early bilinguals’ group, neither the conjunction nor the subtraction analysis provided suprathreshold activation clusters in the comparison between L1 and L2.

##### Late Proficient Bilinguals: Conjunction (L1 ∩ L2) and Subtraction (L1 > L2 and L2 > L1) Analyses

For the late bilinguals’ group, the conjunction analysis showed a shared activation cluster between L1 and L2 in the left (i) inferior frontal gyrus (BA 45).

In the subtraction analyses, neither comparison provided suprathreshold activation clusters.

## Discussion

The present meta-analysis aimed to inspect whether AoA and the traditional classification in early and late bilinguals have an actual role in shaping the bilingual language brain networks, even when accounting for the level of proficiency. We hence identified the two groups of early and late bilinguals (by taking 6 years of age as the AoA cutoff), and also a subgroup of very early bilinguals, in order to investigate the effect of the simultaneous acquisition of two languages. The first preliminary analyses were comprehensive of both the languages the participants knew, as we wanted to obtain a global overview of the whole language network in the three groups. We then performed more focused analyses to assess the functional networks specifically associated with each language, and between- and within-group comparisons between the languages. In this way, we inspected whether, irrespective of other intervening factors, the conventional classification in early and late bilinguals reflected actual differences in the related brain networks. Finally, we replicated these analyses by including only the highly proficient bilinguals, in order to check whether AoA was still relevant when proficiency was comparable (and high) between early and late bilinguals. We carried out these language-specific analyses only for the late bilinguals and for the early bilinguals for which the identification of the first and second language was possible.

### Early, Very Early, and Late Bilinguals

As a first account, we showed that both early and late bilinguals displayed a widespread language network, which was located predominantly in the left (dominant) hemisphere. This network included the classical language areas, together with additional cortical and subcortical regions possibly recruited to support the language functions. For instance, in line with the monolingual language network, both early and late bilinguals activated the classical language areas, such as the Broca’s area (BA 44 and BA 45) known to be involved in a variety of language domains (for reviews see Grodzinsky and Santi, 2008; Friederici, 2011; Price, 2012). Additional shared activations emerged in the left premotor cortex (precentral gyrus) and pre-SMA (posterior-medial frontal gyrus). These regions are involved in articulation-related processes (e.g., Hickok and Poeppel, 2004; Indefrey and Levelt, 2004; Alario et al., 2006; Kemeny et al., 2006), but also in other language tasks, including phonological rehearsal (e.g., Démonet et al., 1992; Paulesu et al., 1993; Awh et al., 1996). However, the role of pre-SMA seems to go beyond these functions to include the control in language use. Actually, Abutalebi and Green (2007, 2016) proposed this area to be one of the stations of the language control network (see afterwards).

Other activation clusters included the middle temporal gyrus—particularly the area associated with the auditory cortex—and fusiform gyrus, both in the left hemisphere. The former is known to be specialized in the perception of words over other non-linguistic sounds (e.g., Binder et al., 1997). The fusiform gyrus is specific for the recognition of words as well, in particular in their written form, across diverse languages and scripts (e.g., Cohen et al., 2000; Turkeltaub et al., 2002; Vigneau et al., 2005; Price and Devlin, 2011). The activation of these two regions might reflect the nature of the stimuli, either auditory or written. Nevertheless, the fusiform gyrus was also shown to contribute to lexical-semantic access, by working in association with the other areas of the middle and inferior temporal gyri (e.g., Papathanassiou et al., 2000; Démonet et al., 2005).

Besides the classical fronto-temporal language areas, also the parietal lobe—particularly the posterior parietal cortex (PPC)—activated in both bilingual groups. This region is not typically devoted to language, although some studies reported its involvement in the performance of some language tasks (for instance in vocabulary learning, see Pasqualotto et al., 2015). Interestingly, this region is relevant to working memory and its activation might, therefore, reflect the heightened necessity for the bilingual speakers to reinforce and elaborate the linguistic information associated with each language (e.g., Gold et al., 2005; Hartwigsen et al., 2010).

Early and late bilinguals also activated the right cerebellum, which is reciprocally connected with the left neocortex and whose involvement in language is becoming progressively more apparent (see for an overview De Smet et al., 2013; Mariën et al., 2014).

Both bilingual groups activated brain areas that more likely reflect the act of having to handle more than one language. In particular, we observed a prominent activation in the DLPFC, which is traditionally associated with high cognitive (executive) functions (e.g., Daffner et al., 2000; McDonald et al., 2000). With respect to bilinguals, DLPFC has been proposed to be a chief station of the network that regulates language selection and control; this region hence modulates the use of each language, for instance by inhibiting the interfering one (i.e., not-in-use; e.g., Abutalebi and Green, 2007, 2016). Another key area of this network has been proposed to be the pre-SMA, which was activated as well, as previously discussed.

An analogous functional interpretation can also be proposed for the insula, which was activated in both hemispheres. Although this region is traditionally viewed as part of the limbic system, its role in language is becoming progressively more evident. Regarding bilingualism, previous evidence supported its involvement in the mechanisms of switching and control (e.g., Wager et al., 2005). Despite the greater role attributed to the left insula, several studies reported bilateral activations in relation to diverse language functions, both receptive and expressive (see the meta-analysis by Oh et al., 2014). Nevertheless, the specific involvement of the right insula requires further investigation. However, it is generally thought to support the dominant hemisphere in various language functions, especially when they become cognitively more demanding (see also the meta-analysis by Vigneau et al., 2011), as can occur in bilingual settings.

Although we did not carry out a direct comparison with the monolingual network (as only a small percentage of studies included data on monolinguals), these preliminary results supported the notion that “the bilingual is not two monolinguals in one person” (Grosjean, 1989); this means that the language network in bilinguals is different from the one that could result from the sum of two language-specific networks in monolinguals (Fabbro, 1999). Actually, bilinguals have to constantly regulate the use of a certain language even when immersed in a monolingual mode, as both languages are, to a certain extent, active (e.g., Marian and Spivey, 2003; Dijkstra, 2005). Even when only one language is in use, there is a continuous interference from the other language, which therefore has to be inhibited (see Paradis, 2004).

The further analysis we conducted investigated the effects associated with an almost concurrent acquisition of the two languages. The functional activations in the very early bilinguals’ group were found in a few areas, such as the left middle temporal gyrus and bilateral cerebella. Interesting was the activation of the left cerebellum, which did not emerge from the previous analyses. This finding leads us to stress once more of the importance of this subcortical structure and hints at a speculative hypotheses for its role (see Ullman, 2006; Paradis, 2009; and Supplementary Material for details).

The fact that very early bilinguals activated in a consistent manner in only a few regions could reflect two possible reasons. First, it is reasonable that these bilinguals need the recruitment of a lower number of regions to perform the language tasks because the very precocious acquisition could imply a lower cognitive effort. This is only a partial explanation, given that the resultant activation clusters did not include other relevant areas of the language network. Hence, this finding may also reflect the low number of studies that have addressed very early language acquisition and, consequently, the low number of provided foci (see the “Materials and Methods” section). These analyses, therefore, need to be replicated once a suitable number of studies is available.

### L1 and L2 Brain Representation

Whereas the previous analyses provided a general overview of the overall brain functioning in response to different AoA, the subsequent analyses were devoted to the investigation of the language brain activations associated with each language. Because a distinction between L1 and L2 in very early bilinguals was rarely possible, we carried out this investigation separately for late bilinguals and for the early bilinguals for which such distinction was achievable.

#### L1

Concerning L1, the results showed different functional networks for early and late bilinguals. With regards to the former, activations (all left-sided) emerged in the classical language areas (i.e., fusiform gyrus, middle temporal gyrus, precentral gyrus, and BA 45) and in regions devoted to cognitive control (i.e., pre-SMA and DLPFC). This suggests that, even in early bilinguals and even when dealing with the first language, there is the need to control and regulate the language use, by possibly suppressing the activation of the second language, which is likely to exert a strong interference.

Regarding late bilinguals, a greater number of activated clusters emerged. These included language-associated areas (as the fusiform gyrus, the middle temporal gyrus, the precentral gyrus, and Broca’s area), control areas (i.e., DLPFC, pre-SMA, and insula in the left hemisphere, and ACC in the right), and the right cerebellum. Also, in late bilinguals, then, language control seems to be required even when performing tasks in L1. This could occur because the first language has to be strongly inhibited in a bilingual context because it tends to prevail even when it is the L2 being used. Consequently, when L1 has to be activated again, great cognitive resources are required to overcome this inhibition, thus implying increased cognitive effort (see switching studies, e.g., Meuter and Allport, 1999; Garbin et al., 2011).

#### L2

Also regarding L2, the network of activations was more substantial for the group of late bilinguals compared to early bilinguals (who activated the superior parietal lobule, precentral gyrus, DLPFC, and pre-SMA), in part probably because of the lower number of contrasts associated with the latter. The late bilinguals’ functional activations were widespread and spanned from the left parietal lobe—both inferior and superior—to the left superior temporal gyrus, frontal regions specifically devoted to language (i.e., BA 45 and pars orbitalis) or control (i.e., left pre-SMA, ACC, and DLPFC, and bilateral insuale), and to the right cerebellum. Some clusters of activation also emerged in the right hemisphere and concerned posterior areas located in the occipital cortex and angular gyrus.

Findings on the number and extent of activations observed for late-learned L2 were not surprising and support the hypothesis of a greater involvement of the areas typically associated with language (e.g., wider activations to compensate for lower efficiency), those devoted to control, and the additional involvement of the right hemisphere. Concerning the activations in the left inferior parietal lobule and in the right hemisphere, a detailed inspection of the contrasts, having concurred to these clusters, helped us hypothesize the rationale for their involvement. In the Supplementary Material, we illustrated these hypotheses and stressed the role that some brain areas, especially in the parietal lobe, can hold in some language functions under specific conditions.

#### Between- and Within-Group Comparison Between L1 and L2

The last analyses we performed were aimed at comparing L1 and L2 within and between each group. For the between-group analysis, L1 did not appear to determine any specific activation for either early or late bilinguals. Similarly, the subtraction analysis did not provide group-specific activations either. However, the main effect results revealed a higher number of activation clusters for the late bilinguals’ group; we cannot exclude this finding to depend on the higher number of available contrasts and the consequent higher probability to draw consistent activations. This limitation prevents us from stating with certainty whether—and in case with which extent—the L1 network might differ as the result of a different AoA. In other words, we cannot comment on the possible feedback impact of this factor on the L1 brain representation (e.g., Titone et al., 2011, for eye-tracking findings).

With regards to the comparison between early and late bilinguals’ L2s, the sole area that activated in conjunction with the two groups, possibly indicating its role irrespective of AoA, was a portion of the left IFG at the border between Broca’s area and DLPFC. The lack of additional common clusters can reasonably reflect the paucity of consistent activations in the group of early bilinguals. In addition, this could also result from actual differences in the L2 activation sites, which can be located in close areas but peak at different coordinates (see Indefrey, 2006). With respect to the direct subtractions between the two groups, the lack of any significant specific activation was not surprising, again possibly resulting from high data variability and consequent lack of consistency. Liu and Cao (2016), who adopted more lenient threshold parameters, reported the findings from a similar subtraction analysis in which they compared the specific L2 activations between early and late bilinguals. Comparable to our analyses, they did not find any region that was more consistently activated in the early bilinguals’ group. They did, however, find a specific late bilinguals’ activation in the region of the left superior frontal gyrus, showing again the greater recruitment of executive control regions as the result of late L2 learning.

Moreover, we can tentatively attribute the lack of consistent results to the inadequacy of the sharp AoA-based classification in reflecting the bilingual brain development. In fact, although development is characterized by clearly defined steps, changes in language appropriation flexibility are not expected to reduce sharply, but rather gradually. In this sense, the re-conceptualization of the critical period in the *sensitive* period could better account for the gradualness of the process (e.g., Flege et al., 1999). In relation to this, it is also important to underline that the critical age for language appropriation was also shown to depend on the language domain. For instance, an early appropriation seems to be more crucial for grammar and phonology/articulation, whereas late appropriation has a less negative impact on lexico-semantics (Paradis, 1994, 2009; Ruben, 1997). In this study, we purposefully investigated the role of AoA on the whole L1 and L2 brain representation, therefore independently from the language domain. A previous meta-analysis, however, inspected the language networks associated with lexico-semantics (Indefrey, 2006), which is probably the most studied domain. This analysis was exploratory as it included a small sample of studies. With the increasing number of neuroimaging studies in bilinguals, in future years it will be possible to have a suitable number of studies in each language domain to investigate the related networks in the two languages.

With regard to the within-group comparisons in the group of the early bilinguals, neither the conjunction nor the subtraction analyses between L1 and L2 provided significant findings. The stringent criteria of paper selection together with the relatively conservative thresholding parameters could possibly explain the discrepancy with Liu and Cao (2016) findings, which showed more relevant L2 activations in the left frontal cortex and insula.

Concerning the late bilinguals, our conjunction analysis revealed L1 and L2 to activate common sites in both classical language areas (i.e., left precentral gyrus and BA 45) and in regions supporting general executive functions (i.e., left pre-SMA and DLPFC). The comparison between the two languages did not provide any regions that appeared to activate selectively for L1. On the contrary, and in line with previously discussed findings, L2 appeared to activate the left pre-SMA and DLPFC more robustly, in a close location to that which emerged from both the conjunction analysis and the same subtraction analysis reported in Liu and Cao (2016).

Trying to interpret these results in the light of the clinical findings on the bilingual patients with aphasia is quite tricky. Clinical literature indeed reports a plethora of different cases, in which the two languages were comparably affected (parallel aphasia) or not (differential aphasia); further, in the latter case, the most affected language could be represented by either L1 or L2.

In this respect, some clinical findings support the role of AoA, by reporting higher impairment in the language that had been learned late (e.g., Diéguez-Vide et al., 2012). Nevertheless, the variety of clinical profiles indicates that many are the factors that contribute to the language brain representation and possible impairment. Among these factors, proficiency and use/exposure have a relevant role in determining which language could be more affected by a clinical event (e.g., Gray and Kiran, 2013). This means that the language that was highly mastered prior to the brain injury is likely to be more resistant to damage, and could, therefore, be better preserved (e.g., Samar and Akbari, 2012). However, AoA has been proposed to retain a leading role, with the role of performance instead emerging only when both languages have been learnt early (see Kuzmina et al., 2019). In the current study, we, therefore, inspected whether, after having accounted for proficiency, AoA could still account for differences between the two languages. In other words, we wanted to assess the actual role of proficiency, which also emerged from a meta-analysis on healthy individuals, in which, however, the role of AoA was not accounted for (Sebastian et al., 2011).

#### Proficient Bilinguals

The last analyses we performed aimed to investigate the language brain representation in early and late bilinguals by removing possible confounding effects due to proficiency. For methodological reasons, we could perform the analyses only on the proficient bilinguals; as long as a high proficiency level was expected to reduce the cognitive effort associated with L2, we inspected whether, proficiency held constant, different brain activations still emerged as the result of different AoA.

These analyses as well were almost exploratory. In fact, the number of experiments included in each analysis was rather low (except for the late bilinguals’ L2) and this was probably the reason why the “classical” language network could not be traced and only a few activation clusters resulted even from the main effect analyses. Nevertheless, this factor, together with the application of stringent thresholds, probably provided the most robust activation clusters for the inspected conditions, which are therefore expected to be highly reliable.

Concerning L1, the main effect analysis showed, in both groups, left-sided activation clusters in areas typically involved in language (in the middle temporal gyrus in early bilinguals and in BA 45 in late bilinguals). Further, the early bilinguals also activated the left pre-SMA. Although our data only allow for speculative interpretations, these results seem to suggest that handling L1 as well requires a certain cognitive control, even when proficiency in both languages is high and appropriation occurred at an early age. This indicates the constant need for bilinguals to monitor and regulate the use of both languages (Abutalebi and Green, 2007, 2016; Grosjean and Li, 2013).

With respect to L2, it is interesting to note that the language brain representation in the late bilinguals was consistently wider than that of the early bilinguals and that the two groups shared a cluster in the left inferior frontal gyrus, at the border between BA 45 and DLPFC. Nevertheless, no one cluster resulted from the direct comparison between the two groups. However, the main effect analysis in the late bilinguals’ group provided activation clusters that did not result from the previous analysis and that corresponded to the bilateral caudate nuclei, one of the regions included in the Abutalebi and Green’s (2007, 2016) language control network. An overall observation, therefore, suggests that, in spite of AoA and proficiency, L2 nevertheless requires the involvement of the executive functions, although the cognitive load appeared to be much greater when appropriation occurred after the age of 6. Late bilinguals, for instance, activated the insula in both hemispheres and control areas such as the pre-SMA. However, we have to remember that these findings might reflect, at least in part, the lower number of contrasts included in the early bilinguals’ analysis.

## Conclusion

Overall, results from these several meta-analyses lead us to conclude that, globally, bilinguals performing language tasks habitually recruit some additional brain regions with respect to the classical language network areas. These additional regions are involved in general cognitive functions, suggesting the constant effort experienced by every bilingual to manage the two languages. Even L1 and every other language possibly acquired since early childhood seem to call for this control. When dealing with two languages, there is clearly a need for their coordination, with the constant inhibition of the not-in-use language (Fabbro, 1999; Abutalebi and Green, 2007; Grosjean and Li, 2013). Nevertheless, in agreement with previous findings, we generally observed that the cognitive effort is stronger for L2, especially when this was learned late (e.g., Indefrey, 2006). The cognitive effort appeared to be present even in proficient bilinguals, although proficiency is likely to reduce the cognitive load associated with late L2 appropriation. This indicates that an early vs. late AoA significantly shapes the bilingual brain, although high proficiency can modulate the languages’ functional representation (Fabbro and Cargnelutti, 2018).

The involvement of general cognitive areas is also, from a clinical viewpoint, a relevant finding. Actually, the cases of differential bilingual aphasia (where one language is more affected than the other) have also been explained in terms of control difficulties (e.g., Verreyt et al., 2013) and rehabilitation programs also focusing on the general cognitive functions were observed to promote language recovery after a brain insult (e.g., Hillis, 2001).

In this study, we did not carry out analyses for the main language domains separately, first because our aim was to identify the most relevant brain regions independently from the assessed task, and, second, because there was not an adequate number of studies to be included in these separate analyses. However, as the different domains are expected to rely more on either AoA (i.e., morpho-syntax and phonology/articulation) or proficiency (i.e., lexico-semantics), future analyses should investigate how these factors modulate the brain representation of these domains.

Further, for reasons we have explained, we found lower-than-expected significant activations in the comparison between the two languages. Intraoperative stimulation mapping studies in bilingual patients showed that the two languages shared many language sites, whereas other sites appeared to be language-specific (e.g., Roux and Trémoulet, 2002; Roux et al., 2004). However, there was a certain inter-subject variability, which could not be attributed uniquely to the different patients’ language history (e.g., AoA or proficiency). These reports result from a clinical condition that might have induced brain reorganization processes and we cannot, therefore, make a direct comparison with our findings. Nevertheless, they suggest the complex interplay between the diverse factors in shaping the language brain representation in bilingual people. Factors such as the linguistic distance between the two known languages, the educational level, and possibly gender could modulate the differential representation of L1 and L2. Future studies should also address proficiency and other relevant parameters, including language exposure and use, in a more thorough way, in order to allow for a reliable assessment of their role. This, in turn, will contribute to a better understanding of the clinical reports of parallel and differential impairment and would, therefore, contribute to the rehabilitation program setting.

## Author Contributions

All the authors contributed to study design, data analysis, result interpretation, and manuscript drafting.

## Conflict of Interest Statement

The authors declare that the research was conducted in the absence of any commercial or financial relationships that could be construed as a potential conflict of interest.
