# Supplementary material for: Language Brain Representation in Bilinguals With Different Age of Appropriation and Proficiency of the Second Language: A Meta-Analysis of Functional Imaging Studies
Source: Front Hum Neurosci. 2019 May 21;13:154. doi: 10.3389/fnhum.2019.00154 (PMC6537025; doi:10.3389/fnhum.2019.00154)
Supplement: Supplementary file 1 [file Data_Sheet_1.pdf]

Supplementary Material

Supplementary Table S1. List of all the studies included in the meta-analyses.

Authors	Year	L1	L2	L2 AoA	L2 proficiency	Technique	Task
*Berken et al.	2015	French (also English for simultaneous bilinguals)	English (for sequential bilinguals)	From birth, for simultaneous bilinguals; 13.9 (5.0), for sequential bilinguals	High	fMRI	Sentence reading
*Buchweitz et al.	2012	Portuguese	English	13.08 (3.1; range: 10-22)	High	fMRI	Inner speech production from different semantic category items
Callan et al.	2014	Japanese	English	At junior high school	Scholastic education	fMRI	Phoneme identification
Callan et al.	2004	Japanese	English	At junior high school	Scholastic education	fMRI	Phoneme identification
Cao et al.	2014	Chinese	English	11.7 (range: 9-13)	Varying	fMRI	Word reading with rhyming judgment
Cao et al.	2013	Chinese	English	11.7 (range: 9-13)	Varying	fMRI	Word reading with rhyming judgment
Chan et al.	2008	Chinese	English	Range: 3-5	Significantly inferior than L1	fMRI	Lexical decision
*Chee et al.	2001	English for Singaporeans; Mandarin for PRC	Mandarin for Singaporeans; English for PRC	At or before 5	High	fMRI	Semantic association between words or characters
*Chee et al.	2000	Chinese	English	At or before 5	High	fMRI	Semantic association between characters
*Das et al.	2011	Hindi (also English for simultaneous bilinguals)	English (for sequential bilinguals)	From birth for simultaneous bilinguals; late (reading started at 10), for sequential bilinguals	Good	fMRI	Word reading

*De Bleser et al.	2003	Dutch or a Flemish dialect	French	At 10	From good to very good	PET	Picture naming
*Ding et al.	2003	Chinese	English	12.17 (range: 11-13)	High	fMRI	Word reading by paying attention to word font or by making semantic categorization
Feng et al.	2015	Chinese	English	12.2 (after 10)	Low-to-intermediate	fMRI	Story reading with comprehension
*Frenck-Mestre et al.	2005	English (also French for early bilinguals)	French (for late)	From birth, for early bilinguals; after 12, for late bilinguals	High	fMRI	Word and sentence reading
Golestani et al.	2006	French	English	At 10-12	Varying	fMRI	Sentence generation from single words
Hernandez et al.	2015	Spanish	English	3.95 (2.17)	Higher than L1	fMRI	Word reading
Hernandez et al.	2007	Spanish for early; English for late	English for early; Spanish for late	4.33 (1.16), for early; 15.83 (2.95), for late	Higher than L1 in early; varying for late	fMRI	Word-gender decision
*Hesling et al.	2012	French	English	11 (0.5)	High for one group, moderate for the other	fMRI	Prosodic speech comprehension
*Jamal et al.	2012	Spanish	English	3.79 (2.21)	Comparable to L1	fMRI	Word reading with attention to word font
Jeong et al.	2015	Japanese	English	11.50 (0.5)	At least intermediate	fMRI	Speech production
Jeong et al.	2011	Japanese	English	11.80 (0.35)	At least intermediate	fMRI	Speech production
*Jeong et al.	2007a	Korean	English	12.3 (1.5)	Level 2 in Society for Testing English Proficiency	fMRI	Sentence comprehension as influenced by syntax
*Jeong et al.	2007b	Chinese or Korean	English	12.1 (1.2), for native	Level 2 in	fMRI	Sentence

				Chinese;<br>12.7 (1.0), for native Korean	Society for Testing English Proficiency		comprehension
*Jiang et al.	2015	Uygur	Chinese	Before 6, for early bilinguals;<br>26.8 (range 20-31), for late bilinguals	Grade 8 in Advanced Chinese Language Test	fMRI	Word reading with semantic judgment
*Kim	2015	Korean	English	After 12	Fluent	fMRI	Picture naming
Kim et al.	2016	Korean	English	8.7 (2.3)	Average	fMRI	Word reading with rhyming judgment
*Klein et al.	1999	Chinese	English	12.1 (range: 10-14)	> 90% accuracy on<br>four different tasks	PET	Noun-verb generation
*Kovelman et al.	2008	Spanish	English	From birth or at 4-5 (at school)	High (> 80% accuracy in screening task)	fMRI	Semantic judgment on sentences with classic or unusual word order
Koyama et al.	2013	Japanese or English	English or Japanese	After 12	Not said explicitly	fMRI	Word reading with phonological matching
*Kumar	2014	Hindi	Urdu	Before 5	Fluent	fMRI	Word reading with attention to word font
Kumar et al.	2010	Hindi	English	Around 8-9	Not said explicitly	fMRI	Phrase reading
Li et al.	2013	Chinese	English	10.64 (2.59)	Moderate	fMRI	Picture naming (plus effect of non-linguistic cues)
Liu et al.	2010	Chinese	English	Around 12	Self-ratings: 5.87/10	fMRI	Picture naming
Luke et al.	2002	Chinese	English	After 10	Varying (self-	fMRI	Syntax and

					ratings: 5.86(0.90)/7 for reading, 5.29(1.60)/7 for speaking)		semantics (reading)
Meschyan & Hernandez	2006	Spanish	English	4.33 (1.16)	Higher than L1	fMRI	Word reading
*Nakada et al.	2001	Japanese or English	English or Japanese	At or after 11	High	fMRI	Sentence reading with comprehension
Nosarti et al.	2009	Italian or English	English or Italian	English: 11 (range: 9-16); Italian: 16 (range: 11-21)	Self-ratings: 75.8(12.1)/100; self-ratings: 54.8(21.3)/100	fMRI	Regular and irregular word reading
*Palomar-García et al.	2015	Spanish	Catalan	From birth or at preschool (range: 0-3)	High, comparable to L1	fMRI	Passive listening; picture naming
Park et al.	2012	Macedonian	English	After 6	High (at least upper intermediate)	fMRI	Lexical decision
*Perani et al.	2003	Spanish or Catalan	Catalan or Spanish	At 3, in kindergarten	High, comparable to L1	fMRI	Word generation (fluency)
*Perani et al.	1998	Experiment 1: Italian	English	After 10	High for one group, low for the other	PET	Story listening with comprehension
		Experiment 2: Spanish or Catalan	Catalan or Spanish	Early childhood, after 2	High	PET	Story listening with comprehension
Perani et al.	1996	Italian	English	After 7	Moderate	PET	Story listening with comprehension
Reiterer et al.	2013	German	English	Around 10	Varying	fMRI	Sentence reading with attention to pronunciation
*Rodriguez-Fornells et	2002	Spanish and Catalan		In the first years of life	High	fMRI	Lexical decision

al.		(no distinction between L1 and L2)					only on Spanish (but not Catalan) words
*Román et al.	2015	Spanish	Catalan	At 3	High	fMRI	Semantic or syntactic judgment on sentences
*Saur et al.	2009	French or German (both for early bilinguals)	German or French (for late bilinguals)	Before 3, for early bilinguals; after 10, for late bilinguals	High	fMRI	Word-order processing in sentences
Suh et al.	2007	Korean	English	At junior high school	Varying	fMRI	Comprehension of sentences with different syntactical difficulty
Sun et al.	2015	Chinese or English	English or Chinese	English: at 12; Chinese: at 18	Self-ratings: 5-6/10	fMRI	Word and character reading
*Tan et al.	2003	Chinese	English	On average at 12	Moderately high	fMRI	Word reading with rhyming task
Tatsuno & Sakai	2005	Japanese	English	At 12	Scholastic education	fMRI	Past-tense verb generation
*van Heuven et al.	2008	Dutch	English	Mean around 11	Self-ratings: 5-6/7	fMRI	Lexical decision in presence of interlingual homographs
*Videsott et al.	2010	Ladin	Italian	At 5	High	fMRI	Picture naming
Vingerhoets et al.	2003	Dutch	French	10.3 (0.5)	Varying	fMRI	Word generation (fluency); picture naming; reading with comprehension
Waldron & Hernandez	2013	Spanish	English	3.18 (1.53; range: 1-6), for early bilinguals; 11 (3.33; range: 7-17), for late bilinguals	Not said explicitly	fMRI	Past-tense verb generation

Yang et al.	2011	Chinese	English	Around 12	Self-ratings: 4-5/7	fMRI	Lexical decision on words and characters
Yokoyama et al.	2006	Japanese	English	11.8 (8-13)	Varying	fMRI	Semantic judgment on sentences with different syntactical difficulty
Yoon et al.	2006	Korean	Chinese (limited to character reading)	Late, at school	Not said explicitly	fMRI	Character reading and picture naming with semantic categorization

Note. If not otherwise indicated, the values related to age of appropriation and proficiency correspond to the mean value for the sample (in parentheses, the standard deviation, when provided).
*: Papers included in the analysis on proficiency; AoA, Age of Appropriation; L1, First language; L2, Second language.

Very early bilinguals and the role of cerebellum

A noteworthy finding for very early bilinguals concerned the specific activation of the left cerebellum. In order to make proper hypotheses on the involvement of this structure, we looked at the contrast having elicited this activation. As well as for the right cerebellum, this activation was prompted by tasks requiring overt articulation, in particular by those involving sentences. It was already demonstrated that some cerebellar districts, for instance lobule VI and crus I (resulting also from our analyses), are devoted to internal motor sequence initiation following the encoding of given phonological information. In agreement with this, lesion studies reported the development of dysarthria after left cerebellar damage (e.g., Lechtenberg and Gilman, 1978). Left-hemisphere lesions were actually observed to be associated with a wide range of language deficits, including the impairment of high-level tasks such as sentence formulation (e.g., Cook et al., 2004; Murdoch and Whelan, 2007).

The cluster of activation we observed in relation to sentence articulation might be attributed to deep sentence processing, phenomenon that probably occurs only in the bilinguals that had been exposed to both languages very early. Actually, late bilinguals are likely to execute strictly the task requirements: If merely required to read the sentences by articulating them, they will probably direct their effort to sole articulation without eliciting further sentence processing (for instance from the semantic viewpoint). This could be supported by findings demonstrating that listening to stories with respect to listening to unrelated sentences determined a greater left cerebellum involvement (e.g., Giraud et al., 2000), suggesting a role also in language comprehension. Alternatively, some other studies noticed that the cerebellar activation increased with practice, through, for instance, increased efficiency in verbal working memory (e.g., Kirschen et al., 2005; Raichle et al., 1994). In line with these findings, it may be suggested this left cerebellum activation to reflect the effect of practice and, therefore, greater language mastery.

L2 network in late bilinguals

Although not resulting from the contrasts analysis, a few interesting activation clusters were observed for L2 in the group of the late bilinguals solely. Among them, the left inferior parietal lobule activation resulted to be primarily elicited by phonological tasks in an L2 that was structurally distant from L1 (e.g., in English for bilinguals with an Asian L1). As this activation spread close to the temporal-parietal junction, we hypothesized a possible a role of this region in managing unfamiliar phonemes.

Noteworthy were also the activations in the right occipital lobe. Also in this case, we checked for the contrasts having concurred to these clusters in order to make reliable assumptions on their involvement. As regards the right primary visual cortex V1, a range of diverse tasks prompted this activation, but only in the bilinguals whose L1 was not an alphabetic language, as the majority of the Indo-European languages are. In these specific cases, L1 was an Asian language, typically characterized by a logographic script. It has been demonstrated that these languages, such as Chinese, normally induce the activation of right-hemisphere regions because they entail a different (e.g., visuo-spatial) elaboration of the stimuli. Hence, in agreement with the assimilation-accommodation hypothesis originally proposed for reading (see Perfetti et al., 2007), it is likely that bilingual speakers with one of these languages as L1 engage for L2 the same functional network developed to process their native language; this could happen in spite of the fact that L2 may require different processing (see Nakada et al., 2001; Nelson et al., 2009; Tan et al., 2003).

With regard to the other two areas that resulted to be activated in the right hemisphere, meaning the middle occipital gyrus and the angular gyrus, a detailed inspection showed that they were recruited during the performance of a wide range of tasks as well, but in particular by those tapping grammar. Especially for what concerned the angular gyrus–and the inferior parietal lobule in general–, the one located in the right hemisphere was largely demonstrated to be involved in visuo-spatial working memory. This cluster however involved also the superior parietal lobule; we

can therefore interpret this finding in light of the clinical studies that observed that lesions affecting this area in either hemisphere impaired the ability to manipulate in memory the verbal information as well, while sparing mere information retrieval (e.g., Koenigs et al., 2009). Taken together, these findings suggest that the recruitment of the parietal lobules occurs to support the performance of non-automatic language tasks. As already widely discussed, it has been postulated that, starting from the age of 6, language abilities begin to be learned via explicit skills, thus resulting in lower language automatization. This is particularly tricky for grammar and phonology. In detail, late AoA is likely to affect the spontaneous application of grammatical rules, at least in terms of automatization. This can thus substantiate the additional activations required to perform grammatical tasks in a late-learned L2.
